# Exploiting Direct Link in Two-Way Half-Duplex Sensor Network over Block Rayleigh Fading Channel: Upper Bound Ergodic Capacity and Exact SER Analysis

**DOI:** 10.3390/s20041165

**Published:** 2020-02-20

**Authors:** Phu Tran Tin, Tan N. Nguyen, Minh Tran, Tran Thanh Trang, Lukas Sevcik

**Affiliations:** 1 IT4Innovations ,VSB-Technical University of Ostrava, 70833 Ostrava, Czech Republic; phutrantin@iuh.edu.vn (P.T.T.); lukas.sevcik@vsb.cz (L.S.); 2 Wireless Communications Research Group, Faculty of Electrical and Electronics Engineering, Ton Duc Thang University, Ho Chi Minh City 70000, Vietnam; 3 Optoelectronics Research Group, Faculty of Electrical and Electronics Engineering, Ton Duc Thang University, Ho Chi Minh City 70000, Vietnam; tranhoangquangminh@tdtu.edu.vn; 4 Faculty of Electrical and Electronics Engineering, Ho Chi Minh City University of Food Industry, 140 Le Trong Tan, Ho Chi Minh City 70000, Vietnam; trangtranthanh1979@gmail.com

**Keywords:** two-way, ergodic capacity (EC), energy harvesting (EH), SER, sensors network

## Abstract

Relay communication, in which the relay forwards the signal received by a source to a destination, has a massive consideration in research, due to its ability to expand the coverage, increase the capacity, and reduce the power consumption. In this paper, we proposed and investigated energy harvesting (EH) based two-way half-duplex (TWHD) relaying sensors network using selection combining (SC) over block Rayleigh fading channel. In this model, we proposed the direct link between two sources for improving the system performance. For the system performance analysis, we investigated and derived the closed-form of the exact and upper bound Ergodic capacity (EC) and the exact form of the symbol error ratio (SER). By using the Monte Carlo simulation, the correctness of the research results is verified in the influence of the main system parameters. From the discussions, we can see that the analytical and simulation agree well with each other.

## 1. Introduction

Radio frequency (RF) energy harvesting (EH) in wireless communications has recently attracted considerable attention which becomes particularly more attractive in applications where battery-limited devices are not easily accessible, and replacing or recharging their batteries is inconvenient, costly and/or unsafe such as devices embedded inside human bodies and wireless sensors operating under dangerous conditions. This solution is based on the fact that RF signals can concurrently carry information and energy, hence allowing energy-constrained nodes to harvest energy and process information simultaneously. This is referred to as simultaneous wireless information and power transfer (SWIPT). Motivated by this, nodes in future wireless networks are envisioned to be energy self-sufficient and more sustainable by harvesting RF signals from the surrounding environment [1,2,3,4,5,6,7,8,9,10,11]. The first basic idea about SWIPT was proposed in [12], in which wireless information and power transfer across a noisy coupled-inductor circuit. After that, other authors have studied the various communication network types based on the SWIPT for different system networks, such as the deployment of PBs for powering a cellular network via MPT investigated in [13], or the communication system network with the single antenna receiver station for energy and information via power splitting protocol studied in [14,15]. In [16], the energy harvesting and information transmission in the wireless multi-antenna communication systems are investigated via a simplified three-node setup. The new wireless RF powered network with the H-AP harvested energy in the downlink and transferred the information in the uplink by TDMA is proposed and demonstrated in [17].

Furthermore, the cooperative relaying network has been deeply studied in the last ten years for improving the system performance of the communication network. The source and destination nodes can be communicated by helping the intermediate relay when the direct link between them is weak or with the long distance. The authors in [18] considered and investigated the outage probability of the three-node cooperative relaying network with a fundamental switching between energy harvesting and data relaying. The relay selection problem in the AF relay network with QoS and harvested energy constraints is proposed in [19]. In this paper, the authors investigated the dependence of the system ergodic capacity and the outage probability on the amount of energy transferred to the RF energy harvesters. In [20], the authors investigated joint wireless information and energy transfer in a two-user MIMO interference channel, in which each receiver either decodes the incoming information data or harvests the RF energy. Further, joint wireless information and energy transfer methods in a general K-user MIMO interference channel was studied in [21]. A wireless cooperative network with multiple source-destination pairs communicate with each other via an energy harvesting relay is presented in [22], and authors investigated the relay’s strategies to distribute the harvested energy among the multiple users and their impact on the system performance. In [23], the authors focused on the two-way amplify-and-forward relaying channels with an energy harvesting relay node. Here, the relay node harvests energy from signals from two sources and uses this energy for information transferring between them with amplify-and-forward mode. For multiple-input multiple-output relay channels [24], proposed a low complexity dynamic antenna switching between information decoding and energy harvesting based on the principles of the generalized selection combiner. The authors in [25] proposed and studied a cooperative multi-hop secured transmission protocol to underlay cognitive radio networks by formulating an effective signal-to-interference-plus-noise ratio (SINR) as well as secrecy capacity under the constraints of the maximum transmit power, the interference threshold, and the hardware impairment level. Furthermore [26], presented a partial relay selection (PRS) protocol to enhance the secrecy performance for cooperative cognitive radio networks (CRNs). For this purpose, the authors investigated the secrecy outage probability (SOP) and the probability of non-zero secrecy capacity (NSC) of the proposed network system. As shown in [27], the problem of designing a good strategy for EH in AF wireless relay systems is considered, and the combination between the wireless power transfer and cooperative jamming (CJ) is studied in [28] for enhancing the physical security in public transportation networks. Full duplex (FD) cognitive radio network, implementing the technique of energy harvesting (EH), is proposed and studied in [29]. And the authors in [30] investigated how to maximize the energy efficiency of the BS while making full use of the relay’s renewable energy and meeting the average throughput requirement. From that point of view, the system performance analysis of the communication cooperative relaying network is the hot research direction in our time.

In this paper, we proposed and investigated Energy Harvesting (EH) based Two-Way Half-Duplex (TWHD) relaying cooperative sensor network using selection combining over block Rayleigh fading channel. Firstly, we proposed the system model with two sources S_1_, S_2,_ and one intermediate relay R. Then, we investigated the system performance in terms of the ergodic capacity (EC) and SER. Finally, all the mathematical analytical expressions are verified by Monte Carlo simulation, and the influence of some main system parameters on the system performance is demonstrated. From the discussions, we can see that the analytical and simulation agree well with each other. The main contribution of this research can be pointed out as the followings:

(1) Energy harvesting based two-way half-duplex relaying cooperative network using selection combining over block Rayleigh fading channel is proposed and investigated
(2) The closed-form of the upper bound EC and exact SER of the model system is derived.
(3) All the results are convinced by Monte Carlo simulation in connection with all primary system parameters.

The rest of this manuscript can be drawn as follows. We provide the system model, the energy and information transfer phases in Section 2. The closed-form expressions of system upper bound EC and SER are derived in Section 3. We introduce the results and some discussions in Section 4. In the last section, some conclusions are proposed.

## 2. Relaying Network Model

**Figure 1:**
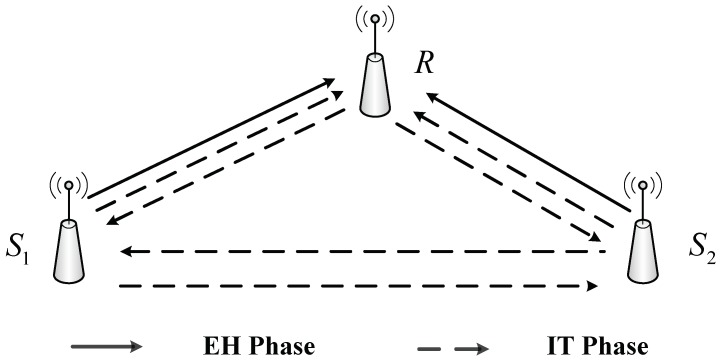
The proposed system model.

**Figure 2:**
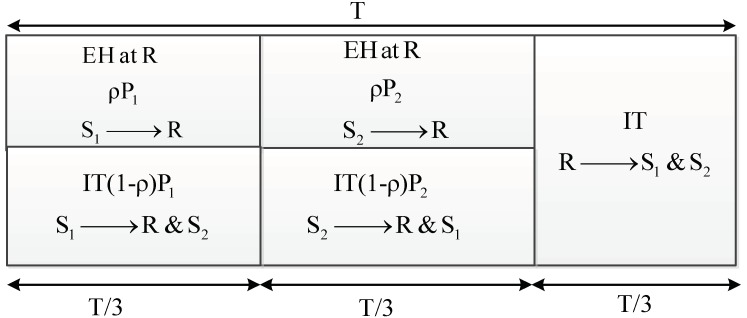
EH and IT in the proposed system.

In this section, the energy harvesting based two-way half-duplex relaying sensor cooperative network is drawn in Figure 1. In this system model, sources are denoted as S_1_ and S_2_, and the relay is *R*. We assume that all links between them are available and are block Rayleigh fading channels. The EH and information transformation (IT) for this proposed model system are illustrated in Figure 2. In this protocol, the transmission is divided into blocks of length T, which consists of three-time slots. In the first time slot T/3, the R harvests energy *ρP*_1_ from the source node S_1,_ and the source uses the energy (1−*ρ*)*P*_1_ for information transmission to R and S_2_ (here 0<ρ<1: is the power splitting factor). In the second interval time T/3, the R harvests energy *ρ*P_2_ from the source node S_2,_ and the source S_2_ uses the energy (1−*ρ*)*P*_2_ for information transmission to R and S_1_. Finally, the remaining time slot T/3 is used for information transferring from the R to the source nodes S_1_ and S_2_ [30,31,32,33,34,35].

### 2.1. Energy Harvesting Phase

Let S_1_ transmits the symbol *x*_1_ in the first phase. The received signal at the relay node R and source node S_2_ can be expressed, respectively, as

(1) $$\begin{array}{l} y_{1,R}^{I}=h_{1,R}x_{1}+n_{r}^{I}\\ y_{1,2}^{I}=h_{1,2}x_{1}+n_{2}^{I} \end{array}$$

where $E\left\{{\left|x_{1}\right|}^{2}\right\}=P_{1},E\left\{•\right\}$ is expectation operator and *P*_1_ represents the average transmit power at the S_1_. Further, $n_{r}^{I}\;\mathrm{and}\;n_{2}^{I}$ denote the zero-mean additive white Gaussian noise (AWGN) with variance N_0_ and $h_{1,R},h_{2,R}$ are the channel gain of S_1_-R and S_2_-R links, respectively.

The harvested energy at the relay node can be given as

(2) $$E_{h}^{I}=\frac{\eta \rho P_{1}{\left|h_{1,R}\right|}^{2}T}{3}$$

where 0<η≤1 is energy conversion efficiency and 0<ρ<1 is the power splitting factor.

In the second phase, the source node S_2_ will transmit the symbol x_2_ to the nodes R and S_1_. Therefore, the received signals at the R and S_1_ can be expressed, respectively, as

(3) $$\begin{array}{l} y_{2,R}^{II}=h_{2,R}x_{2}+n_{r}^{II}\\ y_{2,1}^{II}=h_{2,1}x_{2}+n_{1}^{II} \end{array}$$

where $E\left\{{\left|x_{2}\right|}^{2}\right\}=P_{2}$, P_2_ represents the average transmit power at the S_2_. Further, we assume that $n_{r}^{II}\;\mathrm{and}\;n_{1}^{II}$ are the zero-mean additive white Gaussian noise (AWGN) with variance N_0_.

Similar to the first phase, the total harvested energy at the relay node can be obtained as

(4) $$E_{h}=\frac{\eta \rho \left(P_{1}{\left|h_{1,R}\right|}^{2}+P_{2}{\left|h_{2,R}\right|}^{2}\right)T}{3}$$

We assume that the average transmit power from source S_1_ and S_2_ is equal. So, the Equation (4) can be rewritten as

(5) $$E_{h}=\frac{\eta \rho \left(P_{1}{\left|h_{1,R}\right|}^{2}+P_{2}{\left|h_{2,R}\right|}^{2}\right)T}{3}=\frac{\eta \rho P\left({\left|h_{1,R}\right|}^{2}+{\left|h_{2,R}\right|}^{2}\right)T}{3}$$

where we denote $P_{1}=P_{2}=P$.

Therefore, the average transmit power at the relay node can be given as

(6) $$P_{R}=\frac{E_{h}}{\left(T/3\right)}=\eta \rho P\left({\left|h_{1,R}\right|}^{2}+{\left|h_{2,R}\right|}^{2}\right)$$

### 2.2. Information Transmission Phase

In the first phase, after doing EH, S_1_ will broadcast the information to the R node and S_2_ with remaining power (1−ρ)P. Hence, the received signal at the R node and S_2_ node can be expressed, respectively, as

(7) $$\begin{array}{l} y_{1,R}^{I}=\sqrt{1-\rho }h_{1,R}x_{1}+n_{r}^{I}\\ y_{1,2}^{I}=h_{1,2}x_{1}+n_{2}^{I} \end{array}$$

where $h_{1,2}$ is the channel gain of S_1_-S_2_ link.

Similar to the first phase, the received signal at the R node and S_1_ node can be given in the second phase, respectively, as

(8) $$\begin{array}{l} y_{2,R}^{II}=\sqrt{1-\rho }h_{2,R}x_{2}+n_{r}^{II}\\ y_{2,1}^{II}=h_{2,1}x_{2}+n_{1}^{II} \end{array}$$

where $h_{2,1}$ is the channel gain of S_2_-S_1_ link.

Finally, in the third phase, the received signal at the source S_1_ and S_2_ can be expressed, respectively, as

(9) $$\begin{array}{l} y_{1}^{III}=h_{R,1}x_{R}+n_{1}^{III}\\ y_{2}^{III}=h_{R,2}x_{R}+n_{2}^{III} \end{array}$$

where we denote $E\left\{{\left|x_{R}\right|}^{2}\right\}=P_{R}$. And $h_{R,1},h_{R,2}$ are the channel gain of R-S_1_ and R-S_2_ links, respectively.

In AF technique, in order to ensure that the transmission power at *R* is P_R_, the amplifying coefficient χ can be chosen as

(10) $$\chi =\frac{x_{R}}{y_{R}}=\sqrt{\frac{P_{R}}{(1-\rho )\left[P_{1}{\left|h_{1,R}\right|}^{2}+P_{2}{\left|h_{2,R}\right|}^{2}\right]+N_{0}}}=\sqrt{\frac{P_{R}}{(1-\rho )P\left[{\left|h_{1,R}\right|}^{2}+{\left|h_{2,R}\right|}^{2}\right]+N_{0}}}$$

From Equation (9), the received signal at the source S_1_ can be rewritten as

(11) $$y_{1}^{III}=h_{R,1}\chi y_{R}+n_{1}^{III}=h_{R,1}\chi \left(y_{1,R}^{I}+y_{2,R}^{II}\right)+n_{1}^{III}$$

where $n_{1}^{III}$ denote the zero-mean additive white Gaussian noise (AWGN) with variance N_0_.

Replace Equations (7) and (8) into (11); finally, we have

(12) $$\begin{array}{ll} y_{1}^{III} & =h_{R,1}\chi \left(y_{1,R}^{I}+y_{2,R}^{II}\right)+n_{1}^{III}=h_{R,1}\chi \left[\sqrt{1-\rho }h_{1,R}x_{1}+\sqrt{1-\rho }h_{2,R}x_{2}+n_{r}^{I}+n_{r}^{II}\right]+n_{1}^{III}\\ & =\underset{signal}{\underbrace{\chi h_{R,1}h_{1,R}\sqrt{1-\rho }x_{1}+h_{R,1}\chi \sqrt{1-\rho }h_{2,R}x_{2}}}+\underset{noise}{\underbrace{h_{R,1}\chi n_{r}+n_{1}^{III}}} \end{array}$$

where $n_{r}=n_{r}^{I}+n_{r}^{II}$ denote the total AWGN at the relay with variance N_0_.

This signal contains both messages *x*_1_ and *x*_2_, while the only *x*_2_ is the desired signal at *x*_1_. Since node *x*_1_ perfectly knows its transmitted symbol x_1_, it can eliminate the corresponding self-interference term $\chi h_{R,1}h_{1,R}\sqrt{1-\rho }x_{1}$ from $y_{1}^{III}$. Therefore, Equation (12) can be rewritten as

(13) $$y_{1}^{III}=\underset{signal}{\underbrace{h_{R,1}\chi \sqrt{1-\rho }h_{2,R}x_{2}}}+\underset{noise}{\underbrace{h_{R,1}\chi n_{r}+n_{1}^{III}}}$$

From Equation (13), the signal to noise ratio (SNR) of S_2_-R-S_1_ link can be calculated as

(14) $$\gamma_{2,1}^{AF}=\frac{E\left[{\left|signal\right|}^{2}\right]}{E\left[{\left|noise\right|}^{2}\right]}=\frac{{\left|h_{R,1}\right|}^{2}{\left|h_{2,R}\right|}^{2}P_{2}\chi^{2}(1-\rho )}{{\left|h_{R,1}\right|}^{2}\chi^{2}N_{0}+N_{0}}=\frac{{\left|h_{R,1}\right|}^{2}{\left|h_{2,R}\right|}^{2}P(1-\rho )}{{\left|h_{R,1}\right|}^{2}N_{0}+\frac{N_{0}}{\chi^{2}}}$$

Replace (10) into (14), and after doing some algebra, we have the final form

(15) $$\gamma_{2,1}^{AF}≃\frac{\varphi_{1}\varphi_{2}\psi \eta \rho (1-\rho )}{\eta \rho \varphi_{1}+(1-\rho )}$$

where we denote $\psi =\frac{P_{2}}{N_{0}}=\frac{P}{N_{0}},\varphi_{1}={\left|h_{R,1}\right|}^{2},\varphi_{2}={\left|h_{2,R}\right|}^{2}$.

In the second phase, S_2_ will transmit the data to S_1_ directly, from Equation (8) the received signal destination can be given as

(16) $$\gamma_{2,1}^{direct}=\frac{P_{2}{\left|h_{2,1}\right|}^{2}}{N_{0}}=\psi \varphi_{3}$$

where we denote $\varphi_{3}={\left|h_{2,1}\right|}^{2}$.

Finally, using the selection combining (SC) at the receiver S_1_, the end to end SNR of AF mode at the source S_1_ can be obtained as

(17) $$\gamma_{e2e}^{AF}=\max\left[\gamma_{2,1}^{AF},\gamma_{2,1}^{direct}\right]=\max\left[\frac{\varphi_{1}\varphi_{2}\psi \eta \rho (1-\rho )}{\eta \rho \varphi_{1}+(1-\rho )},\psi \varphi_{3}\right]$$

## 3. Upper Bound Ergodic Capacity and Exact SER Analysis

### 3.1. Upper Bound Ergodic Capacity Analysis

The EC of the proposed system can be formulated as the following

(18) $$C=E\left\{\log_{2}\left(1+\gamma_{e2e}^{AF}\right)\right\}=\int_{0}^{\infty }f_{\gamma_{e2e}^{AF}}(x)\log_{2}(1+x)dx$$

It is easy to observe that Equation (18) is tough to compute in the closed-form expression. So, we will calculate it in the UPPER bound analysis. From Equation (18), EC in UPPER bound can be given as the following equation

(19) $$C=E\left\{\log_{2}\left(1+\gamma_{e2e}^{AF}\right)\right\}\leq C_{UP}$$

where $C_{UP}=\log_{2}\left\{1+E\left\{\gamma_{e2e}^{AF}\right\}\right\}$.

Here, we have

(20) $$E\left\{\gamma_{e2e}^{AF}\right\}=\int_{0}^{\infty }x\times f_{\gamma_{e2e}^{AF}}(x)dx$$

where $f_{\gamma_{e2e}^{AF}}(x)=\frac{\partial F_{\gamma_{e2e}^{AF}}(x)}{\partial x}$.

In order to calculate Equation (20), we have to find $F_{\gamma_{e2e}^{AF}}(x)$. So, combined with Equation (17), $F_{\gamma_{e2e}^{AF}}(x)$ can be given as

(21) $$\begin{array}{ll} F_{\gamma_{e2e}^{AF}}(x) & =\mathrm{Pr}\left(\gamma_{e2e}^{AF}<x\right)=\mathrm{Pr}\left(\max\left[\frac{\varphi_{1}\varphi_{2}\psi \eta \rho (1-\rho )}{\eta \rho \varphi_{1}+(1-\rho )},\psi \varphi_{3}\right]<x\right)\\ & =\underset{P_{1}}{\underbrace{\mathrm{Pr}\left[\frac{\varphi_{1}\varphi_{2}\psi \eta \rho (1-\rho )}{\eta \rho \varphi_{1}+(1-\rho )}<x\right]}}\times \underset{P_{2}}{\underbrace{\mathrm{Pr}\left(\psi \varphi_{3}<x\right)}} \end{array}$$

Firstly, we consider *P*_1_

(22) $$\begin{array}{ll} P_{1} & =\mathrm{Pr}\left\{\frac{\varphi_{1}\varphi_{2}\psi \eta \rho (1-\rho )}{\eta \rho \varphi_{1}+(1-\rho )}<x\right\}=\mathrm{Pr}\left\{\varphi_{2}<\frac{x\left[\eta \rho \varphi_{1}+(1-\rho )\right]}{\varphi_{1}\psi \eta \rho (1-\rho )}\right\}\\ & =\int_{0}^{\infty }F_{\varphi_{2}}\left\{\frac{x\left[\eta \rho \varphi_{1}+(1-\rho )\right]}{\varphi_{1}\psi \eta \rho (1-\rho )}|\varphi_{1}\right\}f_{\varphi_{1}}\left(\varphi_{1}\right)d\varphi_{1}\\ & =\int_{0}^{\infty }F_{\varphi_{2}}\left\{\left[\frac{x}{\varphi_{1}\psi \eta \rho }+\frac{x}{\psi (1-\rho )}\right]|\varphi_{1}\right\}f_{\varphi_{1}}\left(\varphi_{1}\right)d\varphi_{1}\\ & =1-\frac{1}{\lambda_{1}}\exp\left[-\frac{x}{\lambda_{2}\psi (1-\rho )}\right]\times \int_{0}^{\infty }\exp\left[-\frac{x}{\lambda_{2}\varphi_{1}\psi \eta \rho }-\frac{\varphi_{1}}{\lambda_{1}}\right]d\varphi_{1} \end{array}$$

where $\lambda_{1},\lambda_{2}$ are the mean of the random variable (RV) $\varphi_{1},\varphi_{2}$, respectively.

Applying Equation (3.324.1) in [36], Equation (22) can be reformulated as

(23) $$P_{1}=1-2\exp\left[-\frac{x}{\lambda_{2}\psi (1-\rho )}\right]\times \sqrt{\frac{x}{\lambda_{1}\lambda_{2}\psi \eta \rho }}\times K_{1}\left(2\sqrt{\frac{x}{\lambda_{1}\lambda_{2}\psi \eta \rho }}\right)$$

Secondly, P_2_ can be obtained by the following equation

(24) $$P_{2}=\mathrm{Pr}\left\{\psi \varphi_{3}<\gamma_{th}\right\}=\mathrm{Pr}\left\{\varphi_{3}<\frac{\gamma_{th}}{\psi }\right\}=1-\exp\left(-\frac{\gamma_{th}}{\lambda_{3}\psi }\right)$$

where $\lambda_{3}$ is the mean of RV $\varphi_{3}$.

Substituting Equations (23) and (24) into (21), we can obtain $F_{\gamma_{e2e}^{AF}}(x)$ as followings

(25) $$F_{\gamma_{e2e}^{AF}}(x)=\left\{1-\exp\left(-\frac{x}{\lambda_{3}\psi }\right)\right\}\left\{1-2\exp\left[-\frac{x}{\lambda_{2}\psi (1-\rho )}\right]\times \sqrt{\frac{x}{\lambda_{1}\lambda_{2}\psi \eta \rho }}\times K_{1}\left(2\sqrt{\frac{x}{\lambda_{1}\lambda_{2}\psi \eta \rho }}\right)\right\}$$

where $K_{v}(•)$ is the modified Bessel function of the second kind and *v*th order.

Derivate Equation (25) by using the formula $\frac{d}{dx}\left(x^{v}K_{v}(x)\right)=-x^{v}K_{v-1}(x)$, we have

(26) $$\begin{array}{ll} f_{\gamma_{e2e}^{AF}}(x) & =\underset{\Xi (x)}{\underbrace{\frac{\exp\left(-\frac{x}{\lambda_{3}\psi }\right)}{\lambda_{3}\psi }\left\{1-2\exp\left[-\frac{x}{\lambda_{2}\psi (1-\rho )}\right]\times \sqrt{\frac{x}{\lambda_{2}\psi \eta \rho \lambda_{1}}}\times K_{1}\left(2\sqrt{\frac{x}{\lambda_{2}\psi \eta \rho \lambda_{1}}}\right)\right\}}}\\ & +\underset{\Theta (x)}{\underbrace{2\times \exp\left[-\frac{x}{\lambda_{2}\psi (1-\rho )}\right]\times \left\{1-\exp\left(-\frac{x}{\lambda_{3}\psi }\right)\right\}\left\{\begin{array}{l} \frac{1}{\lambda_{2}\psi (1-\rho )}\times \sqrt{\frac{x}{\lambda_{2}\psi \eta \rho \lambda_{1}}}\times K_{1}\left(2\sqrt{\frac{x}{\lambda_{2}\psi \eta \rho \lambda_{1}}}\right)\\ +\frac{K_{0}\left(2\sqrt{\frac{x}{\lambda_{2}\psi \eta \rho \lambda_{1}}}\right)}{\lambda_{2}\psi \eta \rho \lambda_{1}} \end{array}\right\}}} \end{array}$$

Substituting Equation (26) into (20), we have

(27) $$E\left\{\gamma_{e2e}^{AF}\right\}=\int_{0}^{\infty }x\times \Xi (x)dx+\int_{0}^{\infty }x\times \Theta (x)dx$$

The first term of Equation (27) can be derived as

(28) $$\begin{array}{l} \int_{0}^{\infty }x\times \Xi (x)dx=\frac{1}{\lambda_{3}\psi }\int_{0}^{\infty }x\exp\left(-\frac{x}{\lambda_{3}\psi }\right)dx-\frac{2}{\lambda_{3}\psi \sqrt{\lambda_{1}\lambda_{2}\psi \eta \rho }}\times \\ \times \int_{0}^{\infty }x^{3/2}\exp\left\{-x\left[\frac{1}{\lambda_{2}\psi (1-\rho )}+\frac{1}{\lambda_{3}\psi }\right]\right\}\times K_{1}\left(2\sqrt{\frac{x}{\lambda_{2}\psi \eta \rho \lambda_{1}}}\right)dx\\ \;=A_{1}-A_{2} \end{array}$$

where we demote

(29) $$A_{1}=\frac{1}{\lambda_{3}\psi }\int_{0}^{\infty }x\exp\left(-\frac{x}{\lambda_{3}\psi }\right)dx=\lambda_{3}\psi$$

And then applying Equation (6.643,3) in [36], *A*_2_ can be reformulated as

(30) $$\begin{array}{ll} A_{2} & =\frac{2}{\lambda_{3}\psi \sqrt{\lambda_{1}\lambda_{2}\psi \eta \rho }}\int_{0}^{\infty }x^{3/2}\exp\left\{-x\left[\frac{1}{\lambda_{2}\psi (1-\rho )}+\frac{1}{\lambda_{3}\psi }\right]\right\}\times K_{1}\left(2\sqrt{\frac{x}{\lambda_{1}\lambda_{2}\psi \eta \rho }}\right)dx\\ & =\frac{2}{\lambda_{3}\psi \sqrt{\lambda_{1}\lambda_{2}\psi \eta \rho }}\times \frac{\sqrt{\lambda_{1}\lambda_{2}\psi \eta \rho }}{2}\Gamma (3)\Gamma (2)\exp\left\{\frac{1}{2\left[\frac{\lambda_{1}\eta \rho }{\left(1-\rho \right)}+\frac{\lambda_{1}\lambda_{2}\eta \rho }{\lambda_{3}}\right]}\right\}\times {\left[\frac{1}{\lambda_{2}\psi (1-\rho )}+\frac{1}{\lambda_{3}\psi }\right]}^{-2}\\ & \times W_{-2,\frac{1}{2}}\left\{\frac{1}{\left[\frac{\lambda_{1}\eta \rho }{\left(1-\rho \right)}+\frac{\lambda_{1}\lambda_{2}\eta \rho }{\lambda_{3}}\right]}\right\}\\ & =\frac{2\psi }{\lambda_{3}}\times {\left[\frac{1}{\lambda_{2}(1-\rho )}+\frac{1}{\lambda_{3}}\right]}^{-2}\times \exp\left\{\frac{\lambda_{3}(1-\rho )}{2\lambda_{1}\eta \rho \left(\lambda_{3}+\lambda_{2}\left[1-\rho \right]\right)}\right\}\times W_{-2,\frac{1}{2}}\left\{\frac{\lambda_{3}(1-\rho )}{\lambda_{1}\eta \rho \left(\lambda_{3}+\lambda_{2}\left[1-\rho \right]\right)}\right\} \end{array}$$

where Γ(•) is the Gamma function and W(•) is the Whittaker function.

The second term of Equation (27) can be derived as

(31) $$\begin{array}{l} \int_{0}^{\infty }x\times \Theta (x)dx=2\int_{0}^{\infty }x\left\{1-\exp\left(-\frac{x}{\lambda_{3}\psi }\right)\right\}\times \exp\left[-\frac{x}{\lambda_{2}\psi (1-\rho )}\right]\\ \times \left\{\begin{array}{l} \frac{1}{\lambda_{2}\psi (1-\rho )}\times \sqrt{\frac{x}{\lambda_{2}\psi \eta \rho \lambda_{1}}}\times K_{1}\left(2\sqrt{\frac{x}{\lambda_{2}\psi \eta \rho \lambda_{1}}}\right)\\ +\frac{K_{0}\left(2\sqrt{\frac{x}{\lambda_{2}\psi \eta \rho \lambda_{1}}}\right)}{\lambda_{2}\psi \eta \rho \lambda_{1}} \end{array}\right\}dx\\ =B_{1}+B_{2}-B_{3}-B_{4} \end{array}$$

where we denote

$$B_{1}=\frac{2}{\lambda_{2}\psi (1-\rho )\sqrt{\lambda_{1}\lambda_{2}\psi \eta \rho }}\int_{0}^{\infty }x^{3/2}\exp\left\{-\frac{x}{\lambda_{2}\psi (1-\rho )}\right\}\times K_{1}\left(2\sqrt{\frac{x}{\lambda_{1}\lambda_{2}\psi \eta \rho }}\right)dx$$

$$B_{2}=\frac{2}{\lambda_{1}\lambda_{2}\psi \eta \rho }\int_{0}^{\infty }x\exp\left\{-\frac{x}{\lambda_{2}\psi (1-\rho )}\right\}\times K_{0}\left(2\sqrt{\frac{x}{\lambda_{1}\lambda_{2}\psi \eta \rho }}\right)dx$$

$$B_{3}=\frac{2}{\lambda_{2}\psi (1-\rho )\sqrt{\lambda_{1}\lambda_{2}\psi \eta \rho }}\int_{0}^{\infty }x^{3/2}\exp\left\{-x\left[\frac{1}{\lambda_{2}\psi (1-\rho )}+\frac{1}{\lambda_{3}\psi }\right]\right\}\times K_{1}\left(2\sqrt{\frac{x}{\lambda_{1}\lambda_{2}\psi \eta \rho }}\right)dx$$

$$B_{4}=\frac{2}{\lambda_{1}\lambda_{2}\psi \eta \rho }\int_{0}^{\infty }x\exp\left\{-x\left[\frac{1}{\lambda_{2}\psi (1-\rho )}+\frac{1}{\lambda_{3}\psi }\right]\right\}\times K_{0}\left(2\sqrt{\frac{x}{\lambda_{1}\lambda_{2}\psi \eta \rho }}\right)dx$$

By applying Equation (6.643, 3) in [36], we can calculate *B*_1,_ *B*_2_, *B*_3,_ and *B*_4_, respectively, as follows:

(32) $$B_{1}=2\lambda_{2}\psi (1-\rho )\times \exp\left\{\frac{\left(1-\rho \right)}{2\lambda_{1}\eta \rho }\right\}\times W_{-2,\frac{1}{2}}\left\{\frac{\left(1-\rho \right)}{\lambda_{1}\eta \rho }\right\}$$

(33) $$\begin{array}{ll} B_{2} & =\frac{1}{\sqrt{\lambda_{1}\lambda_{2}\psi \eta \rho }}\times \exp\left\{\frac{\left(1-\rho \right)}{2\lambda_{1}\eta \rho }\right\}\times {\left(\frac{1}{\lambda_{2}\psi (1-\rho )}\right)}^{-3/2}\times W_{-\frac{3}{2},0}\left[\frac{\left(1-\rho \right)}{\lambda_{1}\eta \rho }\right]\\ & =\frac{\lambda_{2}\psi {\left(1-\rho \right)}^{3/2}}{\sqrt{\lambda_{1}\eta \rho }}\times \exp\left\{\frac{\left(1-\rho \right)}{2\lambda_{1}\eta \rho }\right\}\times W_{-\frac{3}{2},0}\left[\frac{\left(1-\rho \right)}{\lambda_{1}\eta \rho }\right] \end{array}$$

(34) $$\begin{array}{ll} B_{3} & =\frac{2}{\lambda_{2}\psi (1-\rho )\sqrt{\lambda_{1}\lambda_{2}\psi \eta \rho }}\times \frac{\sqrt{\lambda_{1}\lambda_{2}\psi \eta \rho }}{2}\Gamma (3)\Gamma (2)\\ & \times \exp\left\{\frac{1}{2\left[\frac{\lambda_{1}\eta \rho }{\left(1-\rho \right)}+\frac{\lambda_{1}\lambda_{2}\eta \rho }{\lambda_{3}}\right]}\right\}\times {\left[\frac{1}{\lambda_{2}\psi (1-\rho )}+\frac{1}{\lambda_{3}\psi }\right]}^{-2}\\ & \times W_{-2,\frac{1}{2}}\left\{\frac{1}{\left[\frac{\lambda_{1}\eta \rho }{\left(1-\rho \right)}+\frac{\lambda_{1}\lambda_{2}\eta \rho }{\lambda_{3}}\right]}\right\}\\ & =\frac{2\psi }{\lambda_{2}(1-\rho )}\times {\left[\frac{1}{\lambda_{2}(1-\rho )}+\frac{1}{\lambda_{3}}\right]}^{-3/2}\times \exp\left\{\frac{\lambda_{3}(1-\rho )}{2\lambda_{1}\eta \rho \left(\lambda_{3}+\lambda_{2}\left[1-\rho \right]\right)}\right\}\\ & \times W_{-2,\frac{1}{2}}\left\{\frac{\lambda_{3}(1-\rho )}{\lambda_{1}\eta \rho \left(\lambda_{3}+\lambda_{2}\left[1-\rho \right]\right)}\right\} \end{array}$$

(35) $$\begin{array}{ll} B_{4} & =\frac{2}{\lambda_{1}\lambda_{2}\psi \eta \rho }\times \frac{\sqrt{\lambda_{1}\lambda_{2}\psi \eta \rho }}{2}\times \exp\left\{\frac{1}{2\left[\frac{\lambda_{1}\eta \rho }{\left(1-\rho \right)}+\frac{\lambda_{1}\lambda_{2}\eta \rho }{\lambda_{3}}\right]}\right\}\\ & \times {\left[\frac{1}{\lambda_{2}\psi (1-\rho )}+\frac{1}{\lambda_{3}\psi }\right]}^{-\frac{3}{2}}\times W_{-\frac{3}{2},0}\left\{\frac{1}{\left[\frac{\lambda_{1}\eta \rho }{\left(1-\rho \right)}+\frac{\lambda_{1}\lambda_{2}\eta \rho }{\lambda_{3}}\right]}\right\}\\ & =\frac{\psi }{\sqrt{\lambda_{1}\lambda_{2}\eta \rho }}\times {\left[\frac{1}{\lambda_{2}(1-\rho )}+\frac{1}{\lambda_{3}}\right]}^{-3/2}\times \exp\left\{\frac{\lambda_{3}(1-\rho )}{2\lambda_{1}\eta \rho \left(\lambda_{3}+\lambda_{2}\left[1-\rho \right]\right)}\right\}\\ & \times W_{-\frac{3}{2},0}\left\{\frac{\lambda_{3}(1-\rho )}{\lambda_{1}\eta \rho \left(\lambda_{3}+\lambda_{2}\left[1-\rho \right]\right)}\right\} \end{array}$$

(36) $$C_{UP}=\log_{2}\left[\begin{array}{l} 1+\lambda_{3}\psi -\frac{2\psi }{\lambda_{3}}\times {\left[\frac{1}{\lambda_{2}(1-\rho )}+\frac{1}{\lambda_{3}}\right]}^{-2}\times \exp\left\{\frac{\lambda_{3}(1-\rho )}{2\lambda_{1}\eta \rho \left(\lambda_{3}+\lambda_{2}\left[1-\rho \right]\right)}\right\}\times W_{-2,\frac{1}{2}}\left\{\frac{\lambda_{3}(1-\rho )}{\lambda_{1}\eta \rho \left(\lambda_{3}+\lambda_{2}\left[1-\rho \right]\right)}\right\}\\ +2\lambda_{2}\psi (1-\rho )\times \exp\left\{\frac{\left(1-\rho \right)}{2\lambda_{1}\eta \rho }\right\}\times W_{-2,\frac{1}{2}}\left\{\frac{\left(1-\rho \right)}{\lambda_{1}\eta \rho }\right\}+\frac{\lambda_{2}\psi {\left(1-\rho \right)}^{3/2}}{\sqrt{\lambda_{1}\eta \rho }}\times \exp\left\{\frac{\left(1-\rho \right)}{2\lambda_{1}\eta \rho }\right\}\times W_{-\frac{3}{2},0}\left[\frac{\left(1-\rho \right)}{\lambda_{1}\eta \rho }\right]\\ -\frac{2\psi }{\lambda_{2}(1-\rho )}\times {\left[\frac{1}{\lambda_{2}(1-\rho )}+\frac{1}{\lambda_{3}}\right]}^{-3/2}\times \exp\left\{\frac{\lambda_{3}(1-\rho )}{2\lambda_{1}\eta \rho \left(\lambda_{3}+\lambda_{2}\left[1-\rho \right]\right)}\right\}\times W_{-2,\frac{1}{2}}\left\{\frac{\lambda_{3}(1-\rho )}{\lambda_{1}\eta \rho \left(\lambda_{3}+\lambda_{2}\left[1-\rho \right]\right)}\right\}\\ -\frac{\psi }{\sqrt{\lambda_{1}\lambda_{2}\eta \rho }}\times {\left[\frac{1}{\lambda_{2}(1-\rho )}+\frac{1}{\lambda_{3}}\right]}^{-3/2}\times \exp\left\{\frac{\lambda_{3}(1-\rho )}{2\lambda_{1}\eta \rho \left(\lambda_{3}+\lambda_{2}\left[1-\rho \right]\right)}\right\}\times W_{-\frac{3}{2},0}\left\{\frac{\lambda_{3}(1-\rho )}{\lambda_{1}\eta \rho \left(\lambda_{3}+\lambda_{2}\left[1-\rho \right]\right)}\right\} \end{array}\right]$$

where W(•) is the Whittaker function which can be defined in [36].

### 3.2. SER Analysis

In this section, we obtain new expressions for the SER at the destination. We first consider the outage probability, which was obtained in [31]. Thus, we have

(37) $$SER_{1}=E\left[aQ(\sqrt{2b\gamma_{e2e}^{AF}}\right]$$

$Q(t)=\frac{1}{\sqrt{2\pi }}\int_{t}^{\infty }e^{-x^{2}/2}dx$ is the Gaussian *Q*-function, while *a* and *b* are constants, which are specific for modulation type. (a,b)=(1,1) for binary phase-shift keying (BPSK) and (a,b)=(1,2) for Quadrature Phase Shift Keying (QPSK). As a result, before obtaining the SER performance, the distribution function of $\gamma_{e2e}^{AF}$ is expected. Then, we begin rewriting the SER expression given in Equation (37) directly in terms of outage probability at the source S_1_ by using integration, as follows

(38) $$SER_{1}=\frac{a\sqrt{b}}{2\sqrt{\pi }}\int_{0}^{\infty }\frac{e^{-bx}}{\sqrt{x}}F_{\gamma_{e2e}^{AF}}\left(x\right)dx$$

Substituting Equation (25) into (38), we have

(39) $$\begin{array}{ll} SER_{1} & =\frac{a\sqrt{b}}{2\sqrt{\pi }}\int_{0}^{\infty }\frac{e^{-bx}}{\sqrt{x}}\left[\left\{1-\exp\left(-\frac{x}{\lambda_{3}\psi }\right)\right\}\left\{1-2\exp\left[-\frac{x}{\lambda_{2}\psi (1-\rho )}\right]\times \sqrt{\frac{x}{\lambda_{2}\psi \eta \rho \lambda_{1}}}\times K_{1}\left(2\sqrt{\frac{x}{\lambda_{2}\psi \eta \rho \lambda_{1}}}\right)\right\}\right]dx\\ & =I_{1}-I_{2}-I_{3}+I_{4} \end{array}$$

where we denote

$$I_{1}=\frac{a\sqrt{b}}{2\sqrt{\pi }}\int_{0}^{\infty }\frac{e^{-bx}}{\sqrt{x}}dx,\;I_{2}=\frac{a\sqrt{b}}{2\sqrt{\pi }}\int_{0}^{\infty }\frac{\exp\left[-x\left(b+\frac{1}{\lambda_{3}\psi }\right)\right]}{\sqrt{x}}dx$$

Apply Equation (3.361,2) in [36], *I*_1_ and *I*_2_ can be obtained as, respectively

(40) $$I_{1}=\frac{a}{2}$$

and

(41) $$I_{2}=\frac{a}{2\sqrt{1+\frac{1}{\lambda_{3}b\psi }}}$$

Moreover, *I*_3_ and *I*_4_ can be denoted as

$$I_{3}=\frac{a\sqrt{b}}{\sqrt{\pi \lambda_{1}\lambda_{2}\eta \rho \psi }}\int_{0}^{\infty }\exp\left[-x\left(b+\frac{1}{\lambda_{2}\psi (1-\rho )}\right)\right]\times K_{1}\left(2\sqrt{\frac{x}{\lambda_{2}\psi \eta \rho \lambda_{1}}}\right)dx$$

and

$$I_{4}=\frac{a\sqrt{b}}{\sqrt{\pi \lambda_{1}\lambda_{2}\eta \rho \psi }}\int_{0}^{\infty }\exp\left[-x\left(b+\frac{1}{\lambda_{2}\psi (1-\rho )}+\frac{1}{\lambda_{3}\psi }\right)\right]\times K_{1}\left(2\sqrt{\frac{x}{\lambda_{2}\psi \eta \rho \lambda_{1}}}\right)dx$$

Applying Equation (6.614,4) in [36], *I*_3_ and *I*_4_ can be claimed as, respectively

(42) $$I_{3}=\frac{a\sqrt{b}}{2\sqrt{\pi }}\times \frac{\exp\left(\frac{1}{2\lambda_{1}\lambda_{2}\psi \eta \rho \left[b+\frac{1}{\lambda_{2}\psi (1-\rho )}\right]}\right)}{2\sqrt{\left[b+\frac{1}{\lambda_{2}\psi (1-\rho )}\right]}}\times \Gamma \left(\frac{3}{2}\right)\Gamma \left(\frac{1}{2}\right)\times W_{-\frac{1}{2},\frac{1}{2}}\left(\frac{1}{\lambda_{1}\lambda_{2}\psi \eta \rho \left[b+\frac{1}{\lambda_{2}\psi (1-\rho )}\right]}\right)$$

(43) $$\begin{array}{l} I_{4}=\frac{a\sqrt{b}}{2\sqrt{\pi }}\times \frac{\exp\left(\frac{1}{2\lambda_{1}\lambda_{2}\psi \eta \rho \left[b+\frac{1}{\lambda_{2}\psi (1-\rho )}+\frac{1}{\lambda_{3}\psi }\right]}\right)}{2\sqrt{\left[b+\frac{1}{\lambda_{2}\psi (1-\rho )}+\frac{1}{\lambda_{3}\psi }\right]}}\times \Gamma \left(\frac{3}{2}\right)\Gamma \left(\frac{1}{2}\right)\\ \times W_{-\frac{1}{2},\frac{1}{2}}\left(\frac{1}{\lambda_{1}\lambda_{2}\psi \eta \rho \left[b+\frac{1}{\lambda_{2}\psi (1-\rho )}+\frac{1}{\lambda_{3}\psi }\right]}\right) \end{array}$$

where Γ(•) is the Gamma function and W(•) is the Whittaker function.

Finally, substituting Equations (40)–(43) into (39), SER at the source S_1_ can be obtained as

(44) $$\begin{array}{ll} SER_{1} & =\frac{a}{2}-\frac{a}{2\sqrt{1+\frac{1}{\lambda_{3}b\psi }}}-\frac{a\sqrt{b}}{2\sqrt{\pi }}\times \frac{\exp\left(\frac{1}{2\lambda_{1}\lambda_{2}\psi \eta \rho \left[b+\frac{1}{\lambda_{2}\psi (1-\rho )}\right]}\right)}{2\sqrt{\left[b+\frac{1}{\lambda_{2}\psi (1-\rho )}\right]}}\\ & \times \Gamma \left(\frac{3}{2}\right)\Gamma \left(\frac{1}{2}\right)\times W_{-\frac{1}{2},\frac{1}{2}}\left(\frac{1}{\lambda_{1}\lambda_{2}\psi \eta \rho \left[b+\frac{1}{\lambda_{2}\psi (1-\rho )}\right]}\right)\\ & +\frac{a\sqrt{b}}{2\sqrt{\pi }}\times \frac{\exp\left(\frac{1}{2\lambda_{1}\lambda_{2}\psi \eta \rho \left[b+\frac{1}{\lambda_{2}\psi (1-\rho )}+\frac{1}{\lambda_{3}\psi }\right]}\right)}{2\sqrt{\left[b+\frac{1}{\lambda_{2}\psi (1-\rho )}+\frac{1}{\lambda_{3}\psi }\right]}}\\ & \times \Gamma \left(\frac{3}{2}\right)\Gamma \left(\frac{1}{2}\right)\times W_{-\frac{1}{2},\frac{1}{2}}\left(\frac{1}{\lambda_{1}\lambda_{2}\psi \eta \rho \left[b+\frac{1}{\lambda_{2}\psi (1-\rho )}+\frac{1}{\lambda_{3}\psi }\right]}\right) \end{array}$$

## 4. Numerical Results and Discussion

In this section, we present numerical results to demonstrate the system performance of the proposed system network in the above section. The correctness of the analytical analysis in the above section is verified by the Monte Carlo simulation as in [25,26,27,28,29,36,37,38,39,40,41,42,43,44].

**Figure 3:**
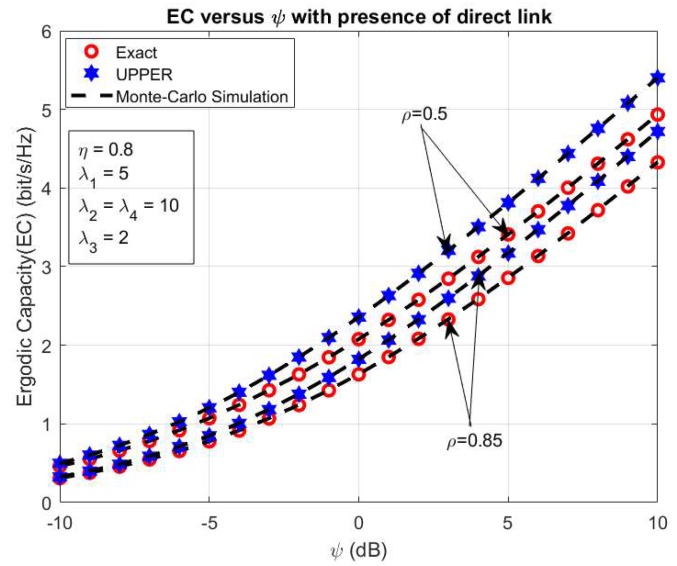
EC versus ψ.

**Figure 4:**
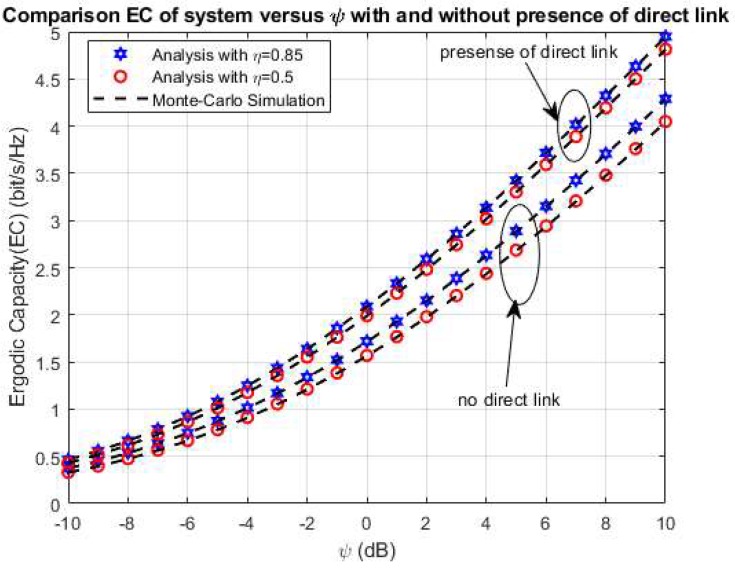
The comparison EC versus ψ.

In this section, we investigate the impact of ψ on the system performance of the proposed system in cases with and without the direct link between the S_1_ and S_2_ nodes. The Figure 3 shows the system EC versus ψ in the presence of the direct link between the S_1_ and S_2_ sources. In Figure 3, we set some primary system parameters as *η* = 0.8, λ_1_ = 5, λ_2_ = λ_4_ = 10, λ_3_ = 2, ρ = 0.5, and 0.85. In this simulation, we consider both the exact and upper bond EC in the influence of ψ as shown in Figure 3. From Figure 3, we can state that both the exact and upper bond system EC rise while we vary ψ from 10 dB to 10 dB and the exact EC is higher than the upper bond system EC with all ψ values. Moreover, the analytical expression of the exact and upper bond system EC in the above section is verified by the simulation results using Monte Carlo Simulation. Furthermore, the comparison system EC in cases with and without a direct link between the sources S_1_ and S_2_ is illustrated in Figure 4 with the primary system parameters as *η* = 0.5 and 0.85, respectively. In the same way with the above case, the system EC significantly increases with rising ψ from 10 dB to 10 dB in both cases with and without a direct link between the sources S_1_ and S_2_ as in Figure 4. In addition, the system EC in the case with a direct link is better than in the case without a direct link between two sources. It can be observed that the direct link can lead to more useful information transmission in the proposed system. In the model system with the direct link, system has two way to transfer the information as via helping relay and direct link. With two way of information transmission via relay and direct link, this case is effective in information transferring in comparison with the case only with helping of the relay as in [45]. Further, the analytical and the simulation results match well for all possible values of ψ as shown in Figure 4.

**Figure 5:**
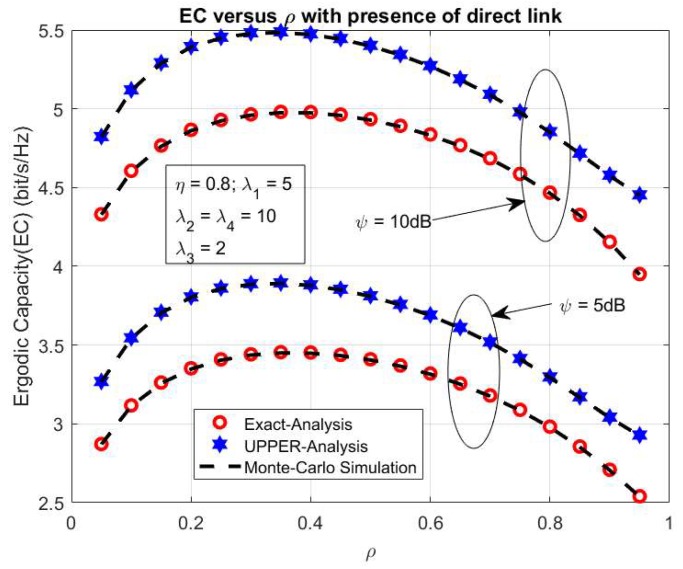
EC versus ρ.

In order to further observe the effect of power splitting coefficient ρ on the exact and upper bond system EC, Figure 5 shows the exact and upper bond system EC versus ρ with the main system parameters as the follows *η* = 0.8, λ_1_ = 5, λ_2_ = λ_4_ = 10, λ_3_ = 2, ψ = 5, and 10 dB, respectively. In this case, we vary the power splitting coefficient ρ from 0 to 1. From the result, we can see that the exact and upper bond system EC increases significantly to the optimal values while ρ increases to 0.4, and after that falls up from the optimal values with the rising of ρ to 1. This is the case because more energy used for the harvesting energy process can lead to an increase of the system EC. Still, the over-harvesting energy process can cause less information transmission and lead to the falling of the system EC as shown in Figure 5. In addition, the difference of the upper and exact maximum ergodic capacity as shown in Figure 3 with ρ = 0.5 is 0.4681 bps/Hz (≈9.5%) 0.4681 bps/Hz (≈9.5%) and ρ = 0.85 is 0.3927 bps/Hz (≈9.1%); and in Figure 5, the difference of the upper and exact maximum ergodic capacity with ψ=5dB is 0.4398 bps/Hz (≈12.7%) and with ψ=10dB is 0.51 bps/Hz (≈10.2%).

**Figure 6:**
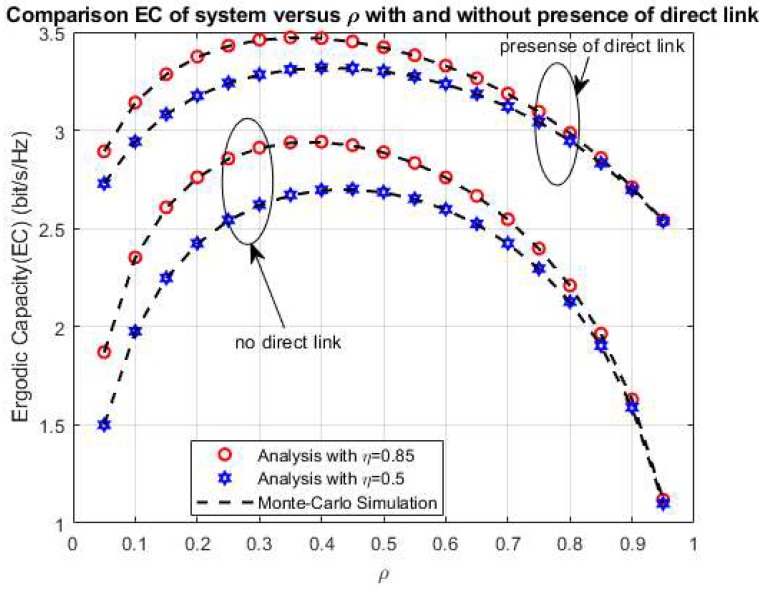
The comparison EC versus ρ.

Furthermore, the comparison of the system EC of the cases with and without a direct link between the sources S_1_ and S_2_ is drawn in Figure 6 with *η* = 0.5 and 0.85, respectively. As shown in Figure 6, the system EC in the case with the presence of the direct link is better than the case without in connection with the better information and energy transmission processes with the direct link between the sources. In both Figure 5 and Figure 6, the simulation and the analytical values are the same with all values of ρ to confirm the analytical analysis in the above section.

**Figure 7:**
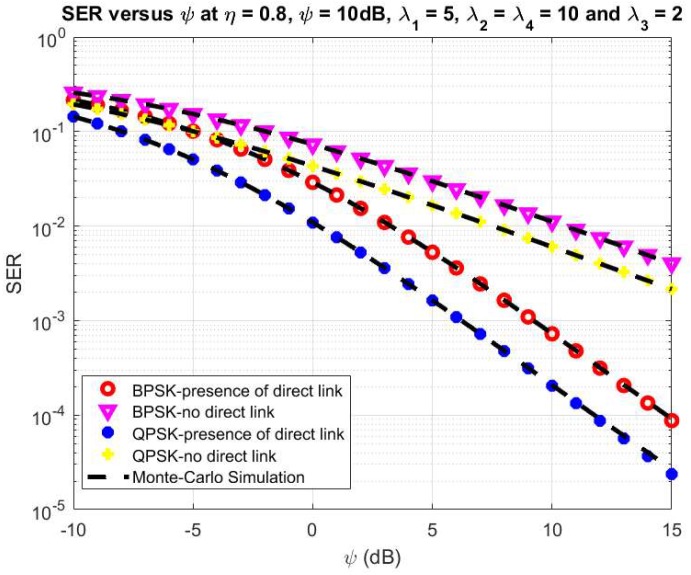
SER versus ψ.

**Figure 8:**
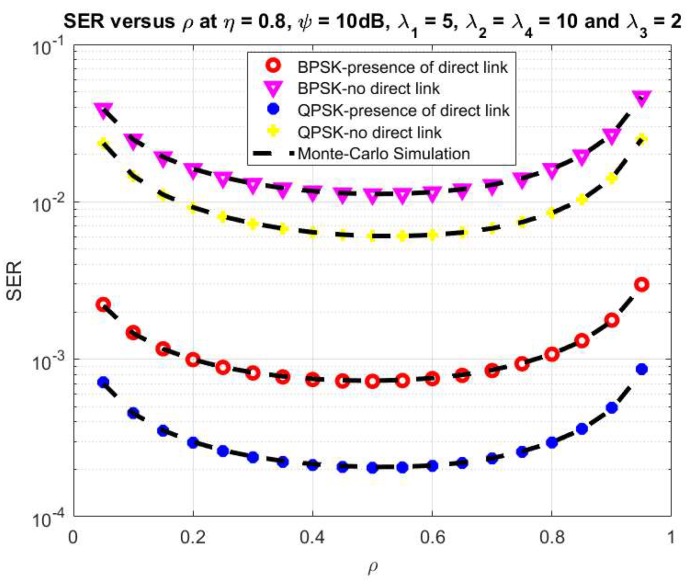
SER versus ρ.

Finally, Figure 7 and Figure 8 show the effect of the ψ and ρ on the system SER, respectively. Here, we set the main system parameters as *η* = 0.8, λ_1_ = 5, λ_2_ = λ_4_ = 10, λ_3_ = 2, ψ = 10 dB for Figure 8 and ρ = 0.5 for Figure 7, respectively. Moreover, the system SER significantly falls while the ψ varies from −10 dB to 10 dB as shown in Figure 7. From the results in Figure 8, it can be seen that the system SER decreases with ρ varies from 0.1 to 0.5, and after that increases with ρ from 0.5 to 1. The optimal value of system SER can be obtained with ρ from 0.4 to 0.6. It can be observed from Figure 6 and Figure 7 that the analytical results have an agreement to the simulations for both cases to verify the correctness of the above analytical section.

## 5. Conclusions

In this paper, we investigated the EH Based TWHD relaying sensor network system using selection combining over block Rayleigh fading channel. In this model, we propose the direct link between two sources for improving the system performance. For the system performance analysis, we investigate and derived the closed-form of the upper bound EC and the exact form of the SER. By using the Monte Carlo simulation, the correctness of the research results is verified in the influence of the main system parameters. From the discussions, we can see that the analytical and simulation agree well with each other. This research can be considered as a novel recommendation for a communication cooperative relaying sensor network.
